# Prevalence of *Streptococcus suis* serotype 2 isolated from pigs: A systematic review and meta-analysis

**DOI:** 10.14202/vetworld.2024.233-244

**Published:** 2024-02-01

**Authors:** Khao Keonam, Nguyen Hoai Nam, Chuleeporn Saksangawong, Patchanee Sringam, Piyawat Saipan, Saijai Kongpechr, Peerapol Sukon

**Affiliations:** 1 Veterinary Science Program, Faculty of Veterinary Medicine, Khon Kaen University, Khon Kaen, 40002, Thailand; 2 Department of Animal Surgery and Theriogenology, Faculty of Veterinary Medicine, Vietnam National University of Agriculture, Trauqui, Gialam, Hanoi, Vietnam; 3 Division of Veterinary Public Health, Faculty of Veterinary Medicine, Khon Kaen University, Khon Kaen, 40002, Thailand; 4 Division of Physiology, Faculty of Veterinary Medicine, Khon Kaen University, Khon Kaen, 40002, Thailand; 5 Division of Anatomy, Faculty of Veterinary Medicine, Khon Kaen University, Khon Kaen, 40002, Thailand; 6 Research Program on Toxic Substances, Microorganisms and Feed Additives in Livestock and Aquatic Animals for Food Safety, Khon Kaen University, Khon Kaen, 40002, Thailand

**Keywords:** meta-analysis, pigs, prevalence, serotype 2, *Streptococcus suis*

## Abstract

**Background and Aim:**

Among *Streptococcus suis* serotypes, *S. suis* serotype 2 is the most significant serotype that causes serious diseases in pigs and humans worldwide. The present study aimed to estimate the global prevalence of *S. suis* serotype 2 isolated from pigs, determine its trend, and explore the factors associated with this serotype.

**Materials and Methods:**

We retrieved relevant published studies from PubMed, Scopus, and the Web of Science. The retrieved citations were screened for possible inclusion. Relevant data were then extracted from the included studies. The random-effects model was used for all meta-analyses. A subgroup meta-analysis was used to assess the heterogeneity of the prevalence for four characteristics (continents, sampling organs, reporting unit, and pig’s health status). A cumulative meta-analysis was performed to determine the cumulative prevalence over time. Meta-regression analysis was used to determine the trend of pooled prevalence of *S. suis* serotype 2 over time.

**Results:**

Of 600 articles retrieved, 36 studies comprising a total sample size of 6939 isolates or samples from 16 countries of four continents were included for meta-analysis. The pooled prevalence of *S. suis* serotype 2 isolated from pigs was 13.6% (95% confidence interval [CI], 10.7%–17.1%), with high heterogeneity among the included studies (Cochran’s Q, 431.6; p < 0.001; I^2^ = 91.9%; Table-1). No statistical significance was observed among subgroups of the four characteristics examined. However, the pooled prevalence of *S. suis* serotype 2 was as high as 16.0% (95% CI, 12.5%–20.3%; n = 16) in diseased pigs compared with 9.9% (95% CI, 5.6%–17.0%; n = 15) in healthy pigs. The pooled prevalence of *S. suis* serotype 2 isolated from pigs did not significantly decrease over time [regression coefficient = −0.020 (95% CI, 0.046–0.006, p = 0.139)]. The pooled prevalence of *S. suis* serotype 2 isolated from pigs fluctuated slightly between 13.2% and 17.8% from 2007 to 2023, although the pooled prevalence gradually decreased from 30.6% in 1987 to over 20% in 2003.

**Conclusion:**

The global prevalence of *S. suis* serotype 2 isolated from pigs was estimated to be 13.6% (approximately 10% in healthy pigs and around 16% in diseased pigs). *S. suis* serotype 2 isolated from pigs did not change significantly over time. These results indicate that *S. suis* serotype 2 remains a problem for the pig industry and poses a threat to human health.

## Introduction

*Streptococcus suis*, which originated from pigs, is an emerging zoonotic pathogen that causes severe illnesses such as meningitis, deafness, septicemia, and even death in humans [1–4]. Although *S. suis* is considered as a commensal bacterium residing mainly in the palatine tonsil and nasal cavity of pigs [5], some strains of *S. suis* can also cause severe diseases, especially in susceptible weaning pigs, resulting in a huge economic loss in the pig industry worldwide [6, 7]. In humans, the burden of *S. suis* infection is highly pronounced in Southeast Asian countries such as Thailand, Vietnam, and Indonesia [8, 9]. Almost all human cases in this region are associated with raw or undercooked pork or pork products [1, 3, 4]. Several sociocultural factors, such as traditional culture, shared beliefs, socioeconomic level, and personal attitudes, play a role in human S. *suis* infection in Southeast Asian countries [10]. In North America and Europe, people typically get infected through close contact with pigs or pork products; therefore, pig farmers and butchers are the occupations at risk [11].

*S. suis* is currently classified into 29 serotypes (1–19, 21, 23–25, 27–31, and 1/2) on the basis of capsular polysaccharide antigens [11–14]. Of these 29 serotypes, *S. suis* serotype 2 has the greatest significance because it can cause severe diseases in both humans and pigs worldwide [1, 11, 13]. *S. suis* serotype 2 is responsible for more than 70% and 25% of clinical cases in humans and pigs, respectively [2, 11, 13]. The prevalence of *S. suis* serotype 2 in healthy and diseased pigs from several regions of the world, including Asia, Europe, and North America [15–25] has been reported. Individual studies are limited in sample size, study location, and study period. Combining individual data (using meta-analytic techniques) from relevant studies would help us to comprehend the global picture of *S. suis* serotype 2 isolated from pigs.

Therefore, the study aimed to use a meta-analysis to estimate the global prevalence and trend of *S. suis* serotype 2 isolated from pigs and to explore some characteristics associated with the heterogeneity of the prevalence.

## Materials and Methods

### Ethical approval

This study was conducted in accordance with the Preferred Reporting Items for Systematic Reviews and Meta-Analyses (PRISMA) statement (http://www.prisma-statement.org/). Ethical approval was not required as no animals or animal-derived products were used in this study.

### Study period and location

The literature search, data collection, and data analysis were conducted at the Faculty of Veterinary Medicine, Khon Kaen University, from June 2022 to July 2023. The included studies were published between 1987 and 2023. The included studies were conducted in 16 countries from Asia, Australia, Europe, and North America.

### Search strategy

Two authors independently performed data search from PubMed, Scopus, and Web of Science to identify the relevant citations reporting the prevalence of *S. suis* serotype 2 isolated from pigs. The search was originally conducted in June 2022 and was updated in May 2023. The PECO structure [26] was used to formulate the research question (P, population = pigs or swine; E, exposure = positive case of *S. suis* infection; C, comparator = negative cases of *S. suis* infection; and O, outcome = the prevalence of *S. suis* isolated from pigs). The most important search keywords were “*swine*, *Streptococcus suis*, prevalence.” For each database, the search was not limited to date but limited to English. The medical subject heading (MeSH) was checked for appropriate and complete keywords. An example search algorithm from the databases was follows: (“swine”[MeSH Terms] OR “swine”[All Fields] OR “swines”[All Fields] OR (“swine”[MeSH Terms] OR “swine”[All Fields] OR “pigs”[All Fields]) OR (“swine”[MeSH Terms] OR “swine”[All Fields] OR “suidae”[All Fields]) OR (“swine”[MeSH Terms] OR “swine”[All Fields] OR (“wart”[All Fields] AND “hogs”[All Fields]) OR “wart hogs”[All Fields]) OR (“swine”[MeSH Terms] OR “swine”[All Fields] OR “warthogs”[All Fields] OR “warthog”[All Fields])) AND “*Streptococcus suis*”[All Fields] AND (“epidemiology”[MeSH Subheading] OR “epidemiology”[All Fields] OR “prevalence”[All Fields] OR “prevalence”[MeSH Terms] OR “prevalance”[All Fields] OR “prevalences”[All Fields] OR “prevalences”[All Fields] OR “prevalent”[All Fields] OR “prevalently”[All Fields] OR “prevalents”[All Fields]).

### Inclusion and exclusion criteria

Two independent reviewers carefully screened the titles and abstracts for initial inclusion after the duplicated citations were removed. Full-text articles that passed the first screening step were carefully examined under specific inclusion and exclusion criteria for final inclusion. This study was included if it reported the prevalence of *S. suis* serotype 2 isolated from pigs. The studies were excluded if (1) they were not related to the prevalence of *S. suis* serotype 2 isolated from pigs, (2) they were reviewed articles, (3) they were case reports, (4) they were experimental studies, and (5) they contained unclear information concerning the prevalence of *S. suis* serotype 2 isolated from pigs. Because *S. suis* serotype 2 is genetically very similar to *S. suis* serotype 1/2, conventional or multiplex polymerase chain reaction (PCR) cannot distinguish between these two serotypes. Therefore, agglutination and coagglutination tests using serotype-specific antisera or additional PCR techniques such as PCR-restriction fragment length polymorphism and whole-genome sequencing data are required for confirmation of *S. suis* serotype 2 [27–29]. Therefore, studies reporting *S. suis* serotype 2 using PCR techniques without additional techniques for serotype confirmation, as mentioned above, were excluded from the study. To evaluate the effect of this exclusion, a sensitivity analysis was performed as described in the statistical analysis section. Disagreements regarding inclusion or exclusion were resolved by discussion.

### Study quality assessment

A checklist for quality assessment adapted from the previous study that had five questions [30]. These were as follows: (1) Was the research objective clearly described and stated? (2) Were the period and location of the study clearly stated? (3) Was the sample categorized into different subgroups? (4) Was the sampling method described in detail? (5) Were the diagnostic techniques clearly pointed out? Scoring of each question was based on a simple scale system (“2” for yes, “0” for no, or “1” for unsure); therefore, a total score for each included study was 10.

### Data extraction

Two authors extracted and verified the data from the included studies. Disagreement regarding data extraction was resolved by discussion. Details of the extracted data were as follows: (1) study identification (name of the first author and publication year), (2) study year, (3) study location, (4) pig’s health status, (5) sampling organs, (6) number of total *S. suis* isolates or samples (sample size), (7) number of *S. suis* serotype 2 positive isolates or samples, and (8) detection methods.

### Statistical analysis

Comprehensive meta-analysis program version 3 (Biostat, Englewood, NJ, USA) was used for all meta-analyses, including overall meta-analysis, subgroup meta-analysis, meta-regression analysis, cumulative meta-analysis, and assessment of publication bias. p < 0.05 was considered statistically significant for all analyses unless otherwise stated. To stabilize the variance of the raw prevalence before pooling, logit transformation was used as follows: logit(p) = ln(p/[1-p]), where “p” is the proportion and “ln” is the natural log [30].

#### Overall meta-analysis

A random-effects model was used to estimate the overall pooled prevalence and its 95% confidence interval (CI). Cochran’s Q and I^2^ statistics were used to assess the heterogeneity. A Cochran’s Q test p < 0.05 implied significant heterogeneity. I^2^ values of 25%, 50%, and 75% were considered low, moderate, and high heterogeneity, respectively [31]. Individual studies were used as a unit of analysis for the overall pooled prevalence.

#### Subgroup meta-analysis

A subgroup meta-analysis was used to assess the heterogeneity of the pooled prevalence of *S. suis* serotype 2 isolated from pre-defined pig characteristics. Subgroup meta-analysis was performed for the following four characteristics: (1) location of the study (among continents), (2) sampling organs (tonsils versus other organs), (3) unit of the report (isolates versus samples), and (4) pig’s health status (healthy versus diseased). For all subgroup meta-analyses, the subgroup within the study was used as a unit of analysis.

#### Meta-regression and cumulative meta-analysis

Meta-regression analysis was used to determine the pooled prevalence trend of *S. suis* serotype 2 isolated from pigs. Meta-regression of the pooled prevalence was performed against the study year. A median of multiple years was used for analysis in a study reporting a multiple-year study. The expected study year was used in a study that did not report the study year. We calculated the expected study year by subtracting 2 years from the publication year (median difference between the publication year and study year of the included studies). A cumulative meta-analysis was also used to determine the cumulative evidence of the pooled prevalence of *S. suis* serotype 2 isolated from pigs over time (the publication year).

#### Sensitivity analysis

Sensitivity analysis was conducted to assess the robustness of the pooled prevalence estimation of *S. suis* serotype 2 infections in pigs. First, we compared the results of the fixed-effects model with those of the random-effects model. Second, we compared the results of using a study as a unit of analysis with those of using a subgroup as a unit of analysis. Third, we compared the results of the included studies with those of the excluded studies reporting *S. suis* serotype 2 but did not describe additional serotype confirmation techniques. Finally, we conducted a leave-one-out meta-analysis to evaluate whether each individually included study influenced the results.

#### Assessment of publication bias

Publication bias was assessed using Begg’s test and Egger’s test with p < 0.1, indicating the presence of publication bias [32, 33]. Publication bias was also visually assessed using a funnel plot. Duval and Tweedie’s trim-and-fill method [34] was used to estimate the adjusted prevalence of *S. suis* serotype 2 isolated from pigs in the case of asymmetrical funnel plots.

## Results

### Identification and selection of studies

**Figure-1:**
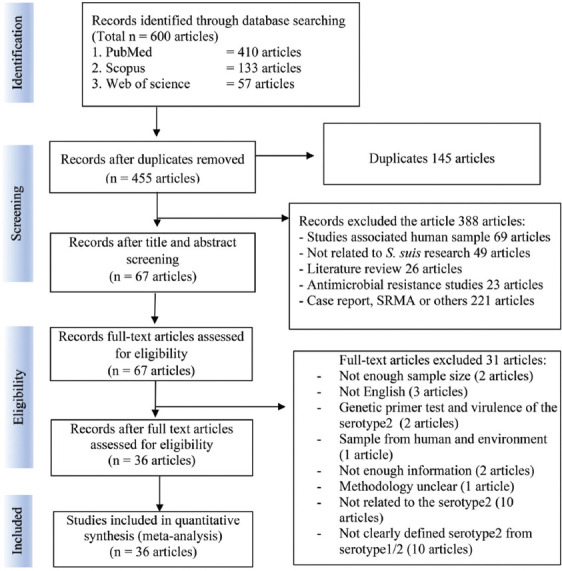
PRISMA flow diagram for the study selection.

A total of 600 articles were identified from three electronic databases (PubMed, 410 articles; Scopus, 133 articles; and Web of Science, 57 articles). Of these, 145 were duplicated. After screening the titles and abstracts, there were 67 full-text articles remaining, of which 388 studies were excluded from the study. This exclusion also included 10 studies reporting *S. suis* serotype 2 but did not describe additional serotype confirmation techniques [35–44]. Ultimately, 36 studies that met the inclusion criteria were included in the meta-analysis [15–25, 45–69]. The study selection process is shown in the flow chart (Figure-1).

### Characteristics of the included studies

Table-1 [15–25, 45–69] summarizes the characteristics of the 36 studies included in the study. Of the 36 included studies, 6939 isolates or samples were examined, resulting in 962 isolates or samples positive for *S. suis* serotype 2. The median sample size was 118 isolates (range, 15–680 isolates). Data were reported from 16 countries from four continents (12 studies from Asia, one study from Australia, 14 studies from Europe, and nine studies from North America) (five studies each from Canada, Spain, and Thailand, four studies from China and the United States, and 13 studies from other countries). Of the 6939 isolates or samples examined, 2032 were from Asia, 2617 from Europe, and 1749 from North America. Of the 6939 isolates or samples examined, 2388 were from healthy pigs, 2973 were from diseased pigs, and 1578 were from pigs with mixed or unspecified health status.

**Table-1:** Characteristics of the included studies.

Reference	Pig’s health status	Country	Study year	Events	Sample size	Prevalence (%)
Aradanas *et al*. [15]	Healthy	Canada	2013–2018	8	90	8.9
	Diseased	Canada	2013–2018	15	183	8.2
Zouharová *et al*. [16]	Diseased	Czech Republic	2018–2022	49	528	9.3
Matiašovic *et al*. [17]	Diseased	Czech Republic	2020–2021	5	39	12.8
Nicholson and Bayles [18]	Unspecified	United States	2015–2017	28	106	26.4
Scherrer *et al*. [19]	Diseased	Switzerland	2006–2019	4	88	4.5
Kerdsin *et al*. [20]	Unspecified	Thailand	2010–2011	39	204	19.1
Bojarska *et al*. [21]	Diseased	Poland, Belarus	2003–2012	21	96	21.9
Niazy *et al*. [22]	Diseased	Canada	NA	8	64	12.5
Lacouture *et al*. [23]	Diseased	Canada	2015–2020	85	680	12.5
Cucco *et al*. [24]	Diseased	Italy	2017–2019	7	78	9.0
Petrocchi-Rilo *et al*. [25]	Diseased	Spain	2019–2020	45	207	21.7
Amass *et al*. [45]	Healthy	United States	1997	0	21	0.0
Baele *et al*. [46]	Unspecified	Belgium	NA	0	60	0.0
Boetner *et al*. [47]	Unspecified	Denmark	1984–1985	33	108	30.6
Brisebois *et al*. [48]	Healthy	Canada	NA	15	164	9.1
Han *et al*. [49]	Healthy	Korea	1999	2	55	3.6
Maneerat *et al*. [50]	Diseased	Thailand	2007	2	24	8.3
	Healthy	Thailand	2007	7	194	3.6
Marois *et al*. [51]	Healthy	France	NA	12	406	3.0
Martinez *et al*. [52]	Mixed	Canada	NA	40	133	30.1
Meekhanon *et al*. [53]	Healthy	Thailand	2014–2015	3	135	2.2
Mogollon *et al*. [54]	Mixed	United States	NA	26	66	39.4
Ngo *et al*. [55]	Healthy	Vietnam	2006–2007	45	317	14.2
Oh *et al*. [56]	Diseased	Korea	2009–2010	36	240	15.0
Paterson *et al*. [57]	Unspecified	Papua New Guinea	NA	126	541	23.3
Sánchez Del Rey *et al*. [58]	Unspecified	Spain	2007–2010	1	320	0.3
Sánchez Del Rey *et al*. [59]	Diseased	Spain	2010–2011	13	15	86.7
	Healthy	Spain	2010–2011	11	128	8.6
Tarradas *et al*. [60]	Healthy	Spain	NA	41	81	50.6
Thongkamkoon *et al*. [61]	Healthy	Thailand	2010	11	196	5.6
Torremorell *et al*. [62]	Diseased	United States	NA	45	242	18.6
van Leengoed *et al*. [63]	Diseased	Netherlands	1983–1985	44	161	27.3
Vela *et al*. [64]	Diseased	Spain	1999–2002	44	302	14.6
Wang *et al*. [65]	Healthy	China	2008–2011	7	61	11.5
Wang *et al*. [66]	Diseased	China	2007–2010	9	26	34.6
	Healthy	China	2007–2010	31	36	86.1
Wongsawan *et al*. [67]	Unspecified	Thailand	2001–2002	5	40	12.5
Zhang *et al*. [68]	Healthy	China	2005–2007	31	421	7.4
Zheng *et al*. [69]	Healthy	China	2011–2012	8	83	9.6

NA=Not available

### Study quality assessment

This study’s quality assessment tool was based on the full 10-point rating scale. The mean ± standard deviation of the overall quality scores of all included studies was 9.03 ± 1.38. Regarding the range of scores from 5 to 10, the median score was 9. All study quality assessment results are presented in Table-2.

**Table-2:** Study quality assessment showing the number of the included studies in each category of the simple rating scale based on a checklist of five items [30].

Items	No. of the included studies		

	Yes	No	Unsure
Was the research objective clearly described and stated?	33	0	3
Was the period and location of the study clearly stated?	25	0	11
Was the sample categorized into different subgroups?	27	4	5
Was the sampling method described in detail?	30	1	5
Was the diagnostic technique clearly pointed out?	35	0	1

#### Overall meta-analysis

The overall pooled prevalence of *S. suis* serotype 2 isolated from pigs was 13.6% (95% CI, 10.7%–17.1%), (n = 36 studies). High heterogeneity was observed among the included studies (Cochran’s Q = 431.6; p < 0.001; I^2^ = 91.9%).

#### Subgroup meta-analysis

**Figure-2:**
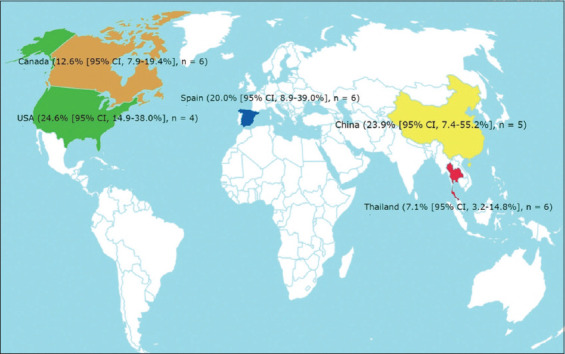
The prevalence estimates of *Streptococcus suis* serotype 2 isolated from pigs from the most reported countries.

The subgroup analysis results are presented in Table-3. No significant difference was observed among subgroups among the four characteristics analyzed. However, the pooled prevalence of *S. suis* serotype 2 was as high as 16.0% (95% CI, 12.5%–20.3%) in diseased and 9.9% (95% CI, 5.6%–17.0%) in healthy pigs. The pooled prevalence of *S. suis* serotype 2 ranged from 12.2% (95% CI, 7.8%–18.6%) in Asia to 16.1% (95% CI, 11.1%–22.8%) in North America. Figure-2 shows the prevalence of *S. suis* serotype 2 isolated from pigs from the most reported countries.

**Table-3:** The overall pooled prevalence of *S. suis* serotype 2 isolated from pigs and subgroup meta-analysis.

Categories	No. of studies or subgroups	Prevalence (%)		Heterogeneity			p-value for subgroup difference
	
		Estimate	95% CI	Q	p-value	I^2^ (%)	
Overall	36	13.6	10.7–17.1	43.6	<0.001	91.9	
Continent^a^							0.631
Asia	14	12.2	7.8–18.6	125.7	<0.001	89.7	
Europe	15	14.1	8.8–21.7	208.8	<0.001	93.3	
North America	10	16.1	11.1–22.8	76.6	<0.001	88.2	
Sampling organs^b^							0.701
Tonsil	20	13.2	8.9–19.0	277.5	<0.001	93.2	
Others	19	14.4	10.9–18.9	143.9	<0.001	87.5	
Unit of report							0.070
Isolates	33	13.6	10.3–17.7	371.2	<0.001	91.4	
Samples	7	19.7	14.6–26.1	31.9	<0.001	81.2	
Pig’s health status^c^							0.114
Healthy	15	9.9	5.6–17.0	218.6	<0.001	93.6	
Diseased	16	16.0	12.5–20.3	89.8	<0.001	83.3	

a Two subgroups were removed from analysis because low number of sample (Australia n = 1, Mixed [Asia-Europe] n = 1).

b One subgroup was removed from analysis due to an unspecified sampling organ.

c Nine subgroups were removed from analysis due to mixed or unspecified health status, CI=Confidence interval

#### Meta-regression and cumulative meta-analysis

**Figure-3:**
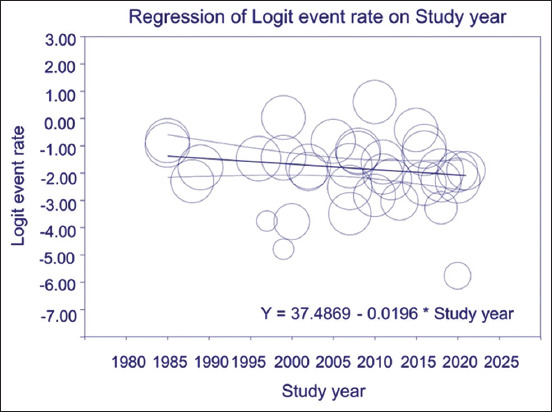
Scatter plot showing results of meta-regression analysis for the correlation between logit event rate (Y-axis) and study year (X-axis). A circle represents each included study (n = 36).

Meta-regression analysis showed that the global prevalence of *S. suis* serotype 2 isolated from pigs did not significantly decrease over time [regression coefficient = −0.020 (95% CI, −0.046–0.006, p = 0.139)]. The meta-regression equation is expressed as the logit event rate = 37.49−0.020 (year), where year is the study year (Figure-3).

**Figure-4:**
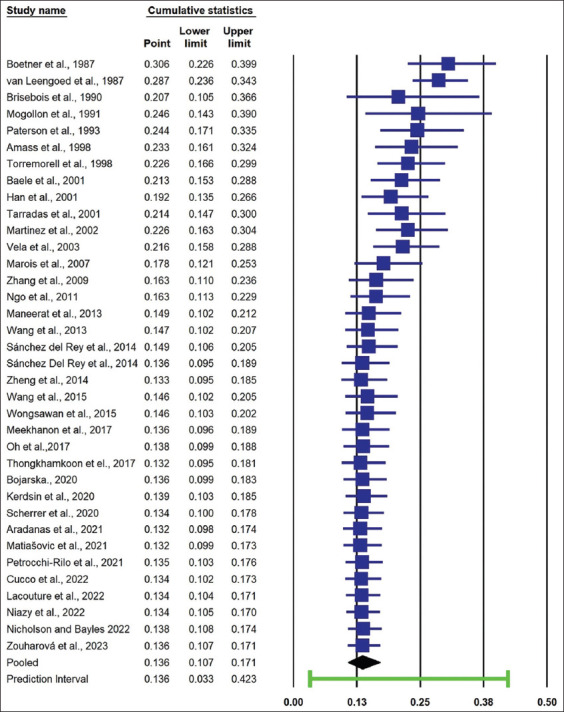
Forest plot of cumulative prevalence and 95% CI of *S. suis* serotype 2 isolated from pigs over time. From 2007 to 2023, the pooled prevalence of *S. suis* serotype 2 isolated from pigs fluctuated between 13.2% and 17.8%.

The cumulative prevalence of *S. suis* serotype 2 isolated from pigs gradually decreased from 30.6% in 1987 to >20% in 2003. From 2007 to 2023, the cumulative prevalence fluctuated between 13.2% and 17.8% (Figure-4).

#### Sensitivity analysis

The sensitivity analysis results are shown in Table-4. The overall pooled prevalence of *S. suis* serotype 2 isolated from pigs in the fixed-effects model was higher than that in the random-effects model (16.9% [95% CI; 15.9%–17.9%] vs. 13.6% [95% CI, 10.7%–17.1%]). Leave-one-out analysis showed that the pooled prevalence of *S. suis* serotype 2 isolated from pigs slightly changed from 12.9% [95% CI, 10.3%–16.0%] to 14.3% [95% CI, 11.4%–17.8%].

**Table-4:** Sensitivity analysis to assess the robustness of the result estimates.

Categories	No. of studies or subgroups	Prevalence (%)	

		Estimate	95%CI
Model			
Fixed effects	36	16.9	15.9–17.9
Random effects	36	13.6	10.7–17.1
Unit of analysis			
Studies	36	13.6	10.7–17.1
Subgroups	40	14.2	11.3–17.1
Serotyping confirmation^a^			
Confirmed	36	13.6	10.7–17.1
Unconfirmed	10	14.1	7.8–24.1
Leave-one-out analysis			
The lowest prevalence^b^	35	12.9	10.3–16.0
The highest prevalence^c^	35	14.3	11.4–17.8

a Specific methods or techniques that used to differentiate *S. suis* serotype 2 from serotype 1/2 were clearly described (confirmed) or (unconfirmed). The unconfirmed 10 studies were excluded from the data synthesis.

b Removed the study of Wang *et al*., [66],

c Removed the study of Marois *et al*., [51], CI=Confidence interval

#### Assessment of publication bias

**Figure-5:**
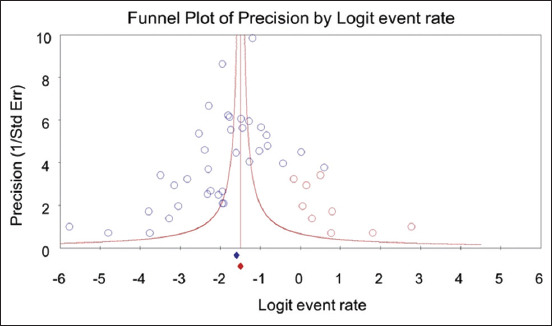
Funnel plot for visual assessment of publication bias. X-axis is the effect size (the logit of the event rate). Y-axis is the precision (standard error). A blue circle represents each included study. A red circle represents an imputed study (or missing study) to make the funnel plot symmetry.

Egger’s test (p = 0.027) and Begg’s test (p = 0.072) indicated publication bias. Funnel plot showed an asymmetrical distribution among the included studies (Figure-5). The trim-and-fill method revealed nine missing (hypothetical) studies. The adjusted point estimate of the overall pooled prevalence of *S. suis* serotype 2 isolated from pigs somewhat increased from 13.6% (95% CI, 10.7%–17.1%) to 18.3% (95% CI, 14.5%–22.8%) after these nine studies were imputed in the model.

## Discussion

Our study synthesized the pooled prevalence of *S. suis* serotype 2 isolated from pigs from 36 included studies worldwide with a total sample size of 6939 isolates or samples. The overall pooled prevalence of *S. suis* serotype 2 isolated from pigs in our study was estimated to be 13.6% (95% CI, 10.7%–17.1%) with high heterogeneity. The point estimate of the pooled prevalence in our study is quite high, indicating that pigs remain a potential source of *S. suis* serotype 2 contamination. The high heterogeneity of the result was within our expectations. Therefore, subgroup meta-analysis and meta-regression were used to assess the heterogeneity or variability of the prevalence among the included studies. Below is our detailed discussion.

Our subgroup meta-analysis did not find a significant difference among the three continents (Asia, Europe, and North America; Australia was excluded from the analysis because it had only one study). The pooled prevalence of *S. suis* serotype 2 isolated from pigs did not significantly differ among continents, ranging from 12.2% (95% CI, 7.8%–18.6%) in Asia, 14.1% (95% CI, 8.8%–21.7%) in Europe, and to 16.1% (95% CI, 11.1%–22.8%) in North America. For all continents, the 95% CI of the point estimate was very wide. This indicated that there was a high variability in the prevalence data from the included studies within each continent. In Asia, the prevalence of *S. suis* serotype 2 in pigs was as low as 2.2% in Thailand [53] and as high as 64.5% in China [66]. In Europe, the prevalence of *S. suis* serotype 2 was as low as 0% [46] in Belgium and as high as 50.6% [60] in Spain. The prevalence of *S. suis* serotype 2 in the United States was as low as 0% in the study of Amass *et al*. [45] and as high as 39.4% in the study of Mogollon *et al*. [54] in North America. This phenomenon has also been observed in other countries, such as Thailand [20, 53], Spain [58, 60], and Canada [15, 52]. The great variability in the prevalence of *S. suis* serotype 2 isolated from pigs among the included studies, even in the same region, reflected the difference in study settings of the included studies and depended on several factors such as coinfection with porcine reproductive and respiratory syndrome virus at weaning, introduction of pigs into the nursery, sow parity, relative humidity, and temperature [7, 70, 71].

Although our study showed that the pooled prevalence of *S. suis* serotype 2 was not significantly different between diseased pigs and healthy pigs, the pooled prevalence was as high as 16.0% in diseased pigs compared with 9.9% in healthy pigs (i.e., 1.6 times higher in diseased pigs). The non-significant result may arise from the small sample size within the subgroup resulting in a low statistical power. Our results indicated that *S. suis* serotype 2 was associated with diseased pigs because the prevalence of *S. suis* serotype 2 in diseased pigs was 1.6 times higher than that in healthy pigs. *S. suis* serotype 2 is known as a virulent strain that causes diseases in humans and pigs worldwide [11, 13]. Clinical manifestations in pigs include septicemia, meningitis, endocarditis, pneumonia, and even death [5, 11]. This impacted the health, welfare, and economic loss of pigs [6]. Most *S. suis* outbreaks in humans result from *S. suis* serotype 2 [1, 11]. Important risk factors for human infections include raw pork consumption, exposure to pigs or pork, pig-related occupation, and male sex [8]. The high pooled prevalence of *S. suis* serotype 2 (9.9%, 95% CI, 5.6%–17.0%; n = 15 subgroups) in healthy pigs was surprising. A high prevalence of *S. suis* serotype 2 in healthy pigs may be a potential source of infection for other pigs and humans [5, 72]. Horizontal transmission primarily occurs through the oro-nasal route or nose-to-nose contact in pigs [5]. In addition, many of the included studies used samples from healthy pigs or pork products from slaughterhouses or wet markets [20, 49, 53, 55, 65, 67]. This production step is close to- and represents a risk for consumers.

For ease of comparison, we divided the sampling organs into two main categories (tonsils vs. other organs). The pooled prevalence of *S. suis* serotype 2 in pigs was not significantly lower in tonsils than in other organs (13.2% and 14.4%, respectively). The palatine tonsil of a pig is a primary organ for the early colonization of *S. suis* [5]. *S. suis* can be found in other organs, such as the lung, brain, spleen, blood, joint fluid, saliva, oral swab, nasal swab, vaginal swab, tongue swab, and pleural effusion [25, 53]. Samples from other organs mainly come from studies of diseased pigs [15, 20, 21, 23, 24, 27, 52, 62]. Because organ collection sampling was confounded by the pig’s health status, further analysis was performed to reveal this confounding effect. Diseased pigs and healthy pigs were separately analyzed on the basis of the sampling organs. The pooled prevalence of *S. suis* serotype 2 was 28.6% (95% CI, 14.8%–48.0%, n = 4) in the tonsil samples and 13.9% (95% CI, 11.0%–17.4%, n = 12) in the other organ samples in diseased pigs. In healthy pigs, the pooled prevalence of *S. suis* serotype 2 was 8.2% (95% CI, 4.1%–15.6%, n = 10) in the tonsil samples and 14.4% (95% CI, 4.0%–40.4%, n = 5) in the other organ samples. These results reveal a confounding relationship between the sampling organs and the pig’s health status.

Most of the included studies reported isolates as a unit of report for the prevalence (n = 33 subgroups) compared with samples as a unit of report for the prevalence (n = 7 subgroups). The pooled prevalence of *S. suis* serotype 2 was not significantly different between isolates and samples (13.6% and 19.7%, respectively). Most of the included studies used isolates as a unit of report because they usually involved identifying all *S. suis* isolate serotype. Therefore, it is more convenient to report each serotype as a proportion of the total isolate. Some included studies [20, 45, 47] reported the prevalence of *S. suis* serotype 2 directly from a positive sample out of a total number of positive samples. A non-significant difference in the pooled prevalence of *S. suis* serotype 2 in the reporting unit indicates that both reporting units are satisfactory.

No significant difference among subgroups in our subgroup analysis does not mean that there is no heterogeneity among subgroups. The number (n) of subgroups in our analysis was small due to the small number of studies included. This may result in a low statistical power and result in a false negative error. If more data are available, we will be able to prove the existence of significant subgroup differences. For example, a significant difference in the pooled prevalence of *S. suis* serotype 2 between diseased pigs and healthy pigs (16.6% [95% CI, 13.2%–20.8%] vs. 9.6% [95% CI, 5.8%–15.4%], respectively) was found if we added ten more studies (that we excluded) reporting *S. suis* serotype 2 but without describing additional techniques for serotype confirmation in our analysis.

Our meta-regression analysis showed that the pooled prevalence of *S. suis* serotype 2 in pigs did not change significantly over time. Our cumulative evidence showed that the pooled prevalence of *S. suis* serotype 2 in pigs fluctuated slightly from 13.2% to 17.8% between 2007 and 2023. Both results indicate that *S. suis* serotype 2 remains a source of problems for pigs and poses a threat to human health. In addition, *S. suis* rapid antimicrobial resistance may exacerbate the problems [73–75].

Sensitivity analysis was used to check the robustness of the decision-making results during the systematic review and meta-analysis. Our sensitivity analysis indicates that the point estimate of the pooled prevalence of *S. suis* serotype 2 in pigs is robust for all categories except the model of choice used in the analysis. The point estimate of the random-effects model (the model of choice of our study) was 13.6%. It was somewhat lower when compared with the fixed-effects model (16.9%). However, the random-effects model was more appropriate in our study because the study settings, sampling designs, and other conditions of the included studies varied greatly [76]. The results of the leave-one-out analysis indicate that individual studies do not distort the point estimate of the pooled prevalence of *S. suis* serotype 2 isolated from pigs. When we removed the study of Wang *et al*. [66] and that of Marois *et al*. [51], the point estimate was the lowest (12.9%) and the highest (14.1%), respectively, from the analysis. These values were very close to the point estimate from all the included studies (13.5%).

Regarding publication bias, Egger’s test (p = 0.027) and Begg’s test (p = 0.072) indicated publication bias in our study. A funnel plot also showed an asymmetrical distribution of the included studies. In addition, the trim-and-fill method revealed nine missing (hypothetical) studies. After these nine hypothetical studies were imputed in the model, the point estimate of the overall pooled prevalence of *S. suis* serotype 2 isolated from pigs increased from 13.6% (95% CI, 10.7%–17.1%) to 18.3% (95% CI, 14.5%–22.8%). This indicates that the pooled prevalence of *S. suis* serotype 2 isolated from pigs would be higher than we estimated without publication bias.

Our study has some limitations. First, our study focused only on the pooled prevalence of *S. suis* serotype 2 because this serotype is very important in human disease. Therefore, the pooled prevalence of all *S. suis* serotypes would be much higher because *S. suis* has 29 specific serotypes [13]. A recent study estimated that the pooled prevalence of *S. suis* in pigs in China during 2000–2021 was as high as 40% [77]. This means that *S. suis* poses a potential threat to the health of pigs and humans. Second, the study locations were mainly from Asia, Europe, and North America. There was only one study from Australia and no study from Africa. Therefore, subgroup analysis for Australia and Africa is not feasible. Interpretation of the global prevalence and subgroup analysis of *S. suis* serotype 2 with regard to the study locations should be carefully performed. Additional information from other regions (if available) may affect the point estimate of the pooled prevalence. Third, we included only studies published in English and retrieved from three databases (PubMed, Scopus, and Web of Science) due to limitations in our research team. Therefore, we may have missed data from other languages, which may have changed the point estimate of the pooled prevalence.

## Conclusion

A random-effects meta-analysis was used to estimate the pooled prevalence of *S. suis* serotype 2 isolated from pigs from 36 included studies containing a total of 6939 isolates or samples from 16 countries of four continents. The overall pooled prevalence of *S. suis* serotype 2 isolated from pigs was 13.6% (95% CI, 10.7%–17.1%) with high heterogeneity among the included studies. The point estimate may be lower than the real-world situation due to the presence of publication bias. The pooled prevalence of *S. suis* serotype 2 was as high as 16.0% in diseased pigs and 9.9% in healthy pigs. There was no significant change in the pooled prevalence of *S. suis* serotype 2 isolated from pigs over time. The results of this study indicate that *S. suis* serotype 2 remains a threat to pigs and humans worldwide.

## Authors’ Contributions

KK, NHN, CS, PaS, PiS, SK, and PeS: Conception. KK and PeS: Extracted, verified, and analyzed the data and drafted and revised the manuscript. All authors have read, reviewed, criticized, and approved the final manuscript.
